# Secondhand Smoke is Associated with Hearing Threshold Shifts in Obese Adults

**DOI:** 10.1038/srep33071

**Published:** 2016-09-08

**Authors:** Yuan-Yung Lin, Li-Wei Wu, Tung-Wei Kao, Chen-Jung Wu, Hui-Fang Yang, Tao-Chun Peng, Yu-Jen Lin, Wei-Liang Chen

**Affiliations:** 1 Department of Otolaryngology–Head and Neck Surgery, Tri-Service General Hospital, National Defense Medical Center, Taipei, Taiwan, Republic of China; 2 Graduate Institute of Medical Sciences, National Defense Medical Center, Taipei, Taiwan, Republic of China; 3 Department of Family and Community Medicine, Division of Family Medicine, Tri-Service General Hospital; School of Medicine, National Defense Medical Center, Taipei, Taiwan, Republic of China; 4 Department of Family and Community Medicine, Division of Geriatric Medicine, Tri-Service General Hospital; School of Medicine, National Defense Medical Center, Taipei, Taiwan, Republic of China

## Abstract

Hearing loss resulted from multiple intrinsic and extrinsic factors. Secondhand smoke (SHS) and obesity had been reported to be related to hearing loss. This study explored the possible associations of SHS and obesity with the hearing threshold. The relations between SHS and the hearing threshold in subjects from three different body mass index classes were analyzed. Our study included data from 1,961 subjects aged 20–69 years that were obtained from the National Health and Nutrition Examination Survey for the years 1999–2004. After adjusting for potential confounding factors, the subjects with the higher tertiles of serum cotinine levels tended to have higher hearing thresholds than those with the lowest tertile of serum cotinine levels (for both trends, p < 0.05). Notably, the obese subjects with the higher tertiles of serum cotinine levels had significantly higher hearing thresholds for high frequencies and low frequencies than those with the lowest tertile of serum cotinine levels (for both trends, p < 0.05). Our study showed a significant positive association between SHS exposure and hearing thresholds in the adult population, especially in obese individuals. Based on our findings, avoiding exposure to SHS, especially in obese adults, may decrease the risk of hearing loss.

Sensorineural hearing loss is an important public health problem with an estimated prevalence of 16.1% in the United States in adults aged 20 to 69 years^1^. When the aging of the population is taken into consideration, the prevalence can only be expected to increase^2^. Hearing loss can be a disabling condition that reduces the quality of life. Loss of hearing negatively affects cognitive and emotional status, work productivity and social interaction, leading to psychosocial problems such as depression, progressive isolation and withdrawal. Hearing loss can be result from multiple intrinsic and extrinsic factors which can be further classified as cochlear aging, environmental (e.g., noise exposure, ototoxic medications), genetic predisposition and health co-morbidities (e.g., tobacco smoking, hypertension, diabetes)^2^,^3^. Although aging and noise exposure are the leading causes of hearing loss in adults, other factors such as secondhand smoke (SHS) and obesity are also associated with loss of hearing^4^,^5^,^6^,^7^.

SHS is an important public health concern. SHS exposure increases the risk of cardiovascular disease to approximately 30% in nonsmoking individuals^8^. In the literature, there was a mountain of evidence regarding to smoking and hearing loss, but a little about the association between SHS and hearing loss. An active smoker is personally responsible for his/her own toxic exposures; however, involuntary exposures via SHS may place never smokers at increased risk for hearing loss^4^,^5^,^9^,^10^. A study by Fabry *et al*. examined a nationally representative cross-sectional dataset of 3307 individuals and found that SHS was significantly related with hearing loss in nonsmokers^4^. It merits a further investigation into the effect of SHS on hearing impairment.

Obesity is associated with increased risks of disease, disability, and death^11^. Obesity is considered to be a multifactorial metabolic disorder. Cardiovascular disease, cerebrovascular disease, hypertension, and diabetes are related with obesity. An association between obesity and hearing impairment has been observed in epidemiologic studies^6^,^11^,^12^. A multicenter European study of 4083 subjects found that higher body mass index (BMI) was related with hearing loss at both high frequencies and low frequencies^12^. In addition, the Nurses’ Health Study II of 116,430 women found that a higher BMI (≧40) and a larger waist circumference (WC; >88 cm) were associated with an increased risk of hearing loss^11^.

As these previous studies have shown, there is evidence indicating that SHS and obesity are related to hearing impairment. Moreover, these two distinct factors seem to share some possible mechanisms causing hearing impairment, such as atherosclerosis^13^, oxidative stress^2^,^14^,^15^,^16^,^17^, and inflammation^16^,^17^. It is reasonable to hypothesize that SHS and obesity together may have a combined effect that impairs hearing. From the viewpoint of public health, this investigation can help to set up more effective health education and public promotion campaigns to reduce SHS exposure and to introduce weight loss programs. The purpose of our study was to investigate the possible associations of SHS and obesity with the hearing threshold. We used data from the National Health and Nutrition Examination Survey (NHANES). In particular, we evaluated whether the relation between SHS and the hearing threshold was different for individuals with three different BMIs.

## Results

### Characteristics of the study population

Our study subjects were 1,961 participants from the NHANES 1999–2004 sample. The demographic distribution and clinical characteristics of our study group are presented according to the tertiles of the serum cotinine level (Table 1). Among the subjects, the mean age was 39.72 years (SD = 13.06) and 36.9% of the subjects were men. In the subjects with higher tertiles of serum cotinine concentration, WC, BMI, and uric acid levels were significantly higher, and age and HDL-C level were significantly lower; there were more males and fewer non-Hispanic whites.

### Association between serum cotinine level and hearing thresholds

The results of multiple linear regression analysis based on serum cotinine tertiles are shown in Table 2. Subjects in the higher tertiles of serum cotinine level tended to have a higher hearing threshold. In model 1, model 2, and model 3, positive associations between serum cotinine level and the hearing thresholds (high-PTA and low-PTA) were observed. In model 3, after subsequent adjustment with other covariates, the β coefficients of the high-PTA comparing the second and third tertiles of serum cotinine levels to the lowest tertile of serum cotinine levels were 0.012 and 0.049, respectively (p value for the trend = 0.002); the β coefficients of the low-PTA were −0.010 and 0.041, respectively, for the similar comparison of tertiles (p value for the trend = 0.013).

### Associations among serum cotinine level, BMI and hearing thresholds

The analyses of the relations between the serum cotinine level, BMI and hearing thresholds are presented in Table 3. Obese subjects in the higher tertiles of serum cotinine levels had a higher hearing threshold. Among the three models, significant positive associations between the serum cotinine level and the hearing thresholds (high-PTA and low-PTA) were observed. In model 3, in obese subjects the β coefficients of the high-PTA comparing the second and third tertiles of serum cotinine levels to the lowest tertile of serum cotinine levels were 0.042 and 0.076, respectively (p value for the trend = 0.005); the β coefficients of the low-PTA were 0.028 and 0.068, respectively, for the similar comparison of tertiles (p value for the trend = 0.017).

## Discussion

We studied a large and nationally representative group of nonsmoking adults in the United States who were exposed to SHS. On the basis of serum cotinine levels and self-reported information and consistent with a dose-dependent effect, we found that study subjects with the higher tertiles of serum cotinine levels had significantly higher high-frequency and low-frequency hearing thresholds compared with those with the lowest tertile of serum cotinine levels. Notably, this association was only observed in the obese subjects and was not found in normal weight subjects or overweight subjects. Among the obese subjects, those with higher tertiles of serum cotinine levels had significantly higher hearing thresholds than those with the lowest tertile of serum cotinine levels; this relation was not observed in the normal weight subjects or the overweight subjects. In other words, SHS did not seem to have a significant association with a hearing threshold shift in normal weight subjects or overweight subjects, but did have a significant positive association with a hearing threshold shift in obese subjects.

Previous studies have shown a correlation between SHS and hearing loss or cochlear auditory dysfunction^4^,^5^,^9^,^10^,^20^,^21^,^22^,^23^. In addition, our study results are in accord with the findings from previous studies that show a dose-response effect between the level of SHS exposure and the risk of hearing loss^5^,^10^,^23^. Cruickshanks *et al*. reported an association between smoking and hearing loss and also described a significant correlation between SHS and hearing loss. Nonsmokers who lived with a household member who smoked had a higher risk of hearing loss than those who did not live with a smoker (odds ratio, 1.94; 95% confidence interval, 1.01–3.74; P = 0.047)^9^. In a cross-sectional study of 164,770 adults aged between 40 and 69 years in the United Kingdom, Dawes *et al*. found that SHS exposure was associated with increased odds of hearing loss and a dose-response effect was observed^5^. Either prenatal or postnatal SHS exposure had a damaging effect on cochlear function in children. In mothers with prenatal SHS exposure, there were damaging effects on cochlear auditory function in neonates, as assessed by otoacoustic emissions testing^21^, and moreover, the increased risks of hearing loss still existed in adolescence and these risks were higher than in adolescents without prenatal SHS exposure^22^. Postnatal SHS exposure in children also had a negative effect on cochlear auditory function and increased the risk of hearing loss^10^,^20^,^23^.

The exact mechanism of ototoxicity due to smoking is not clear. It has been proposed that smoking may be deleterious to the inner ear due to a direct ototoxic effect of nicotine or another toxin or to vascular ischemia or hypoxemia in the cochlea from vasospasm, vasoconstriction, increased blood viscosity, production of carboxyhemoglobin, or blood vessel arteriosclerosis^13^,^24^,^25^,^26^,^27^. Oxidative stress caused by smoking also affects auditory function^2^,^14^,^15^. A previous study found nicotinic-like receptors in the cochlear hair cells, which may suggest that tobacco smoke has detrimental effects on hair cell function due to a possible action during the auditory neurotransmission^28^. Considering these possible mechanisms either collectively or separately, smokers or individuals exposed to SHS may be more susceptible to hearing impairment than nonsmokers or individuals not exposed to SHS. Furthermore, the impact of smoking could interact with other factors or with detrimental auditory exposures such as noise, causing synergic harmful effects on auditory function^2^,^7^,^29^,^30^.

Although obesity-related co-morbidities can impair hearing, obesity itself may have some specific associations with hearing impairment. In an animal study, obesity could exacerbate the hearing impairment with elevated hearing thresholds^31^. In humans, central obesity (i.e., either a higher amount of visceral adipose tissue or a greater WC), was found to be related to an elevated hearing threshold^32^,^33^. There is also evidence indicating that oxidative stress is a critical factor that links obesity with its associated complications^16^.

As far as we know, mechanisms of age-related hearing loss include hypoxia, atherosclerosis, inflammation, oxidative stress and production of reactive oxygen species, mitochondrial dysfunction, and subsequent apoptosis of the cochlear cells^2^. The two distinct factors, SHS and obesity, seem to share some possible mechanisms accelerating the process of hearing impairment, such as atherosclerosis^13^, oxidative stress^2^,^14^,^15^,^16^,^17^, and inflammation^16^,^17^. This might be the explanation why they have synergistic actions that impair hearing. Obesity and smoking-related atherosclerosis may lead to lumen narrowing in the internal auditory artery and reduce cochlear blood flow^13^. Reduced blood circulation to the cochlea, whether of microvascular or macrovascular origin, can cause dysfunction of the stria vascularis, diminished perfusion and hypoxia of cochlear hair cells and neurons, and can subsequently impact hearing function^34^,^35^,^36^. There is growing evidence that suggests that oxidative stress and inflammation are the important underlying factors connecting obesity with its related complications^16^, both of which could be aggravated by cigarette smoking^17^. A similar result was also observed in an animal study in which tobacco smoking was associated with worsening of the oxidative stress and subsequent chronic kidney injury in obese animals^37^. In a study that estimated the SHS exposure by measuring the nicotine level in hair, there was a significant relation between SHS exposure and oxidative stress in overweight subjects and obese subjects^38^. In other words, SHS exposure in obese individuals may worsen the existent oxidative stress and inflammation, which also could be exacerbated by hypoxia (associated with obesity and smoking-related atherosclerosis)^2^. They may synergistically cause hearing impairment. However, it needs further longitudinal study and laboratory research to investigate whether SHS and obesity have a synergistic effect that impairs hearing or not.

The tonotopic organization of the cochlea results that hair cells at the base of the cochlea respond to high frequencies and those at the apex to the low frequencies^39^. In our study analysis, we observe a significant positive association between secondhand smoke exposure and hearing thresholds of both high-frequency and low-frequency in the adult population of the United States, especially in obese individuals. The threshold elevation was across the frequency spectrum, affecting the cochlea from the base to the apex. Schuknecht characterized four categories of age-related hearing loss based on clinical and histopathologic changes within the cochlea: sensory, neural, strial, and cochlear conductive^40^. He described “strial” (also known as metabolic) hearing loss pathologically based on atrophy of the stria vascularis microvasculature throughout the cochlea. Individuals with this type of hearing loss tended to have relatively flat audiograms, i.e. similar losses in hearing sensitivity across the frequency range. We found that secondhand smoke exposure produce a similar broad-spectrum pattern of hearing impairment, like observations in other reports^4^,^10^, and this effect may be mediated by involvement of the stria vascularis. Although our results fit with that hypothesis, further study is needed to clarify whether the stria vascularis is the exact site of global cochlear injury.

Our study had several limitations. First, it is a cross-sectional study in which the serum cotinine levels and the hearing thresholds were measured at one timepoint, rather than a longitudinal study. Thus, causal inferences cannot be made. Second, no information regarding congenital or genetic hearing loss was available in the NHANES dataset. Third, a recall bias in the medical history data cannot be excluded. Fourth, our analysis dates were obtained from the 1999–2004 NHANES, so our present study focused on the participants aged 20–69. The interpretation of our results should be cautiously. Finally, misclassification of SHS was possible because the exposure estimate was based on the serum cotinine level that has an average half-life of 17 hours.

## Conclusions

Many epidemiologic studies about evaluating the possible risk factors of hearing impairment were conducted to find the preventable factors. They can give us inspiration of further laboratory research and an improved understanding of the etiologic risk factors related to hearing impairment which results in significant public health benefits. Noise-induced hearing loss is by far the most important preventable cause of hearing loss^2^,^3^,^12^. Other modifiable factors include smoking, ototoxic medication (aminoglycoside antibiotics, loop diuretics, cisplatin, or anti-inflammatory agents), diabetes, hypertension, and hyperlipidaemia^2^,^3^,^5^,^12^. Educating and advising patients to maintain good general health and fitness would have benefits on hearing preservation. Our study showed a significant positive association between SHS exposure (as assessed by higher serum cotinine levels) and hearing thresholds in the adult population of the United States, especially in obese individuals. In spite of previous studies showing a relation between SHS and hearing loss, our study is the first study to further indicate that SHS and obesity may have a synergic effect on hearing impairment. Based on our findings, avoiding exposure to SHS, especially in obese adults, may reduce the risk of hearing loss.

## Methods

### Study population

The NHANES is a large cross-sectional study that is designed to represent the non-institutionalized civilian population in the United States. This survey is annually conducted by the National Center for Health Statistics of the CDC. The survey includes an initial household interview and subsequent physical examinations and laboratory tests that are performed at a mobile examination center (MEC) to collect pertinent information.

We limited our study subjects to include NHANES participants who were nonsmokers exposed to SHS, as defined by both the personal questionnaire during the household interview and the self-reported questionnaire regarding smoking status that was administered at the MEC, as well as the nicotine level measured by a laboratory test. Our study examined data from 1,961 subjects aged 20–69 years that were obtained from the NHANES for the years 1999–2004 and included the following: demographic data, results of physical examinations, laboratory tests, questionnaire contents, and audiometric testing. Our study excluded individuals with current or ever occupational noise exposure or with incomplete data regarding either SHS exposure or other variables assessed in this study.

### Audiometric measures

According to the NHANES protocol for 1999–2004, after the initial household interview and medical examination, half of the subjects were randomly scheduled for audiometric testing. Subjects who were eligible for testing were excluded if they could not take off their hearing aids or could not tolerate the auditory headphones. Audiometric testing was performed in the MEC sound-isolated room by technicians who were trained by a certified audiologist from the National Institute for Occupational Safety and Health. The audiometric instrumentation contained an interacoustics model AD226 audiometer equipped with standard TDH-39P headphones and EARTone 3A insert earphones. Both ears were tested across the frequencies of 500, 1,000, 2,000, 3,000, 4,000, 6,000, and 8000 Hz with an intensity range from −10 to 120 dB to measure the air-conduction pure tone hearing thresholds. The high-frequency pure-tone average (high-PTA) was calculated as the average of the pure-tone hearing level at 3,000, 4,000, 6,000, and 8,000 Hz. The low-frequency pure-tone average (low-PTA) was calculated as the average of pure tone hearing level at 500, 1,000, and 2,000 Hz. High-PTA and low-PTA were used for evaluation the high-frequency and low-frequency hearing impairment, respectively^4^,^10^. We chose the worse ear with the lower pure-tone average, for the regression analysis. More details of audiometric measures are available at http://www.cdc.gov/nchs/data/nhanes/au.pdf.

### Definition of SHS exposure

Cotinine, a major proximal metabolite of nicotine, is extensively used as a biomarker of exposure to cigarette smoke for either active smoking or SHS^18^. A nonsmoker was defined as an individual who: (1) according to the personal questionnaire, had not smoked at least 100 cigarettes or had not smoked a pipe or a cigar or used snuff or chewing tobacco at least 20 times in their entire life and (2) and according to the MEC tobacco questionnaire, had not used tobacco or nicotine products in the past 5 days. Nonsmokers with serum cotinine levels that were detectable but <10 ng/mL were defined as having been exposed to SHS^19^. Due to the improvement of laboratory methods over time, the limit of detection (LOD) for cotinine was 0.050 ng/mL in the NHANES data for 1999–2000, but 0.015 ng/mL in the NHANES data for 2001–2004. However, for uniformity in using the 1999–2004 NHANES data, we used the LOD from the 1999–2000 NHANES data (0.050 ng/mL) for all individuals. For analysis purposes for values below the LOD, we used the equation of the LOD divided by the square root of two (0.035 ng/mL).

### Definition of obesity

We classified subjects based on BMI according to the WHO definition, as follows: a BMI ≧ 30 is obese, a BMI of 25 to <30 is overweight, a BMI of 18.5 to <25 is normal weight, and a BMI < 18.5 is underweight. The BMI is defined as the body mass (in kilograms) divided by the square of the body height (in meters) and is expressed in units of kg/m^2^.

### Assessment of covariates

Demographic data that included age, sex, race/ethnicity, status of smoking, and personal history were collected. Race was categorized as non-Hispanic black, non-Hispanic white, or other. Both the personal questionnaire and the self-reported questionnaire were used to determine smoking status. Participants were defined as having diabetes mellitus (DM) if they self-reported a diagnosis by a physician, had a fasting glucose level of ≥126 mg/dL, had a random glucose level of ≥200 mg/dL, or used diabetic medications. Heart disease was considered to be present if participants had experienced or had been diagnosed with angina, myocardial infarction, coronary artery disease, or congestive heart failure. Stroke was determined from self-reports. The current use of ototoxic medications including aminoglycosides, loop diuretics, non-steroidal anti-inflammatory drugs, or anticancer drugs, was ascertained from self-reports. Abnormal otoscopy was determined if there were abnormal results of an initial otoscopic screening examination of the eardrum and external acoustic canal before the audiometric testing. An audiometric impedance tympanometer was used for the tympanometry. Abnormal tympanometry was determined if the compliance was ≦0.3 ml or the middle-ear peak pressure was ≦−150 daPa. Body measurements, including height, weight, and WC (measured to the nearest 0.1 cm at the top of the hip bone after breathing out normally) were recorded from participants at the MEC. Uric acid data were determined using the Hitachi 737 analyzer. Levels of high-density lipoprotein cholesterol (HDL-C) were enzymatically measured using the Hitachi 704 analyzer. All measurements used standardized methods with documented accuracy that followed the CDC guidelines. All detailed information regarding biospecimen collection and processing protocols are available at the NHANES website.

### Statistical analyses

SPSS (version 18.0 for Windows; SPSS, Inc., Chicago, IL, USA) was used to conduct the statistical analyses. Two-sided p-values < 0.05 were considered to indicate significant differences. Some covariates including age, WC, BMI, uric acid, and HDL-C were treated as continuous variables and were presented as the mean value ± standard deviation (SD). Some covariates such as sex and race were regarded as categorical variables and were presented as numbers with percentages. A log transformation was performed to normalize the distribution of the PTA hearing thresholds. Our study used tertile-based analysis that divided the serum cotinine level into tertiles with the individuals in the lowest tertile considered as the reference group. The three tertiles of the serum cotinine level were as follows: T1 < 0.0226 ng/mL, 0.0226 = T2 < 0.0719 ng/mL, and 0.0719 = T3 < 10 ng/mL. Linear regression analysis was used to assess the association of the tertiles of serum cotinine level with hearing threshold. A multiple-model approach was constructed for adjustments of covariates. Model 1 was adjusted for age, gender and race. Model 2 was model 1 after further adjustment for WC, BMI, HDL-C, and uric acid. Model 3 was model 2 after further adjustment for abnormal otoscopy and tympanometry, current use of ototoxic medications, history of DM, heart disease or stroke. The p-values for the trend tests were determined by treating the tertile of the serum cotinine level as a continuous variable (ranging from 1 to 3) to assess the associations across the increase in the tertile of the serum cotinine level with the PTA hearing threshold.

### Ethics statement

The National Center for Health Statistics Institutional Review Board approved the NHANES study, and written informed consent was received from all participants prior to the study. However, our study was exempt from IRB review because we used a publicly available, unidentifiable dataset.

## Additional Information

**How to cite this article**: Lin, Y.-Y. *et al*. Secondhand Smoke is Associated with Hearing Threshold Shifts in Obese Adults. *Sci. Rep.* **6**, 33071; doi: 10.1038/srep33071 (2016).

## Figures and Tables

**Table 1:** Characteristics of study participants.

Variables	Tertile of serum cotinine level (ng/mL)			P value
	Tertile 1 (<0.0226) (n = 639)	Tertile 2 (0.0226–<0.0719) (n = 655)	Tertile 3 (0.0719–<10) (n = 667)	
**Continuous variables, mean** ± **SD**				
Age (year)	40.16 ± 12.81	40.69 ± 13.56	38.06 ± 12.64	<0.001
Waist circumference (cm)	94.94 ± 14.79	94.00 ± 15.16	97.58 ± 16.35	<0.001
BMI (kg/m^2^)	28.08 ± 6.25	28.04 ± 6.37	29.63 ± 7.06	<0.001
Uric acid (mg/dL)	4.78 ± 1.35	4.94 ± 1.35	5.25 ± 1.49	<0.001
HDL-C (mg/dL)	57.02 ± 16.48	53.40 ± 15.38	53.72 ± 15.69	<0.001
Worse ear				
Low-PTA (dB)	12.79 ± 10.28	12.50 ± 9.93	13.76 ± 10.78	0.069
High-PTA (dB)	20.05 ± 16.45	21.35 ± 17.07	20.88 ± 16.41	0.361
Log low-PTA	1.01 ± 0.29	1.00 ± 0.31	1.03 ± 0.31	0.037
Log high-PTA	1.18 ± 0.33	1.20 ± 0.35	1.21 ± 0.33	0.333
**Categorical variables, n (%)**				
Male	184(28.8)	244(37.3)	296(44.4)	<0.001
Race				
Non-Hispanic white	319(49.9)	270(41.2)	250(37.5)	<0.001
Ever had diagnosis				
Diabetes	45(7.1)	35(5.4)	41(6.2)	0.455
Heart disease	12(1.9)	14(2.1)	15(2.3)	0.887
Stroke	6(0.9)	9(1.4)	8(1.2)	0.765
Ototoxic medication (present use)	39(6.1)	58(8.9)	49(7.3)	0.168
Abnormal otoscopy	141(22.1)	102(15.6)	117(17.5)	0.008
Abnormal tympanometry	62(10.6)	59(9.9)	56(9.1)	0.709

Definition of abbreviations. SD = standard deviation. BMI = body mass index. HDL-C = high-density lipoprotein cholesterol. Low-PTA = Pure tone average at low frequencies. High-PTA = Pure tone average at high frequencies.

**Table 2:** Regression coefficients of tertiles of cotinine level for hearing threshold.

		High-PTA				Low-PTA			
Variables		Unadjusted	Model 1	Model 2	Model 3	Unadjusted	Model 1	Model 2	Model 3
Tertile of serum cotinine level									
Tertile 1	β^*^ (95%CI)	Reference	Reference	Reference	Reference	Reference	Reference	Reference	Reference
	P value	—	—	—	—	—	—	—	—
Tertile 2	β (95%CI)	0.021 (−0.016,0.058)	0.005 (−0.027,0.037)	0.010 (−0.021,0.042)	0.012 (−0.019,0.044)	−0.016(−0.049,0.017)	−0.015 (−0.047,0.017)	−0.013 (−0.045,0.020)	−0.010 (−0.042,0.022)
	P value	0.257	0.756	0.530	0.442	0.348	0.357	0.444	0.548
Tertile 3	β (95%CI)	0.026 (−0.010,0.063)	0.045 (0.013,0.076)	0.049 (0.017,0.081)	0.049 (0.017,0.081)	0.026 (−0.006,0.059)	0.042 (0.010,0.075)	0.040 (0.008,0.073)	0.041 (0.008,0.073)
	P value	0.161	0.006	0.003	0.002	0.114	0.010	0.016	0.014
P for trend		0.163	0.006	0.003	0.002	0.109	0.010	0.015	0.013

Adjusted covariates: Model 1 = age, sex, race.

Model 2 = Model 1 + (BMI, HDL-C, uric acid, waist circumference).

Model 3 = Model 2 + (abnormal otoscopy, abnormal tympanometry, present use of ototoxic agents, history of diabetes, heart disease, stroke).

Definition of abbreviations: High-PTA = Pure tone average at high frequencies, Low-PTA = Pure tone average at low frequencies, BMI = body mass index, HDL-C = high-density lipoprotein cholesterol.

*β Coefficients can be interpreted as differences in hearing threshold comparing subjects in the upper two cotinine level quartiles to those in the lowest quartile.

**Table 3:** Regression coefficients of tertiles of cotinine level and body mass index for hearing threshold.

Variables	Tertile of serum cotinine level (ng/mL)	High-PTA						Low-PTA					
		Normal weight		Overweight		Obesity		Normal weight		Overweight		Obesity	
		β (95%CI)	P value	β (95%CI)	P value	β (95%CI)	P value	β (95%CI)	P value	β (95%CI)	P value	β (95%CI)	P value
Model 1	Tertile 1	Reference	—	Reference	—	Reference	—	Reference	—	Reference	—	Reference	—
	Tertile 2	−0.031 (−0.086,0.025)	0.280	0.014 (−0.040,0.069)	0.607	0.038 (−0.016,0.092)	0.173	−0.039 (−0.095,0.016)	0.167	−0.024 (−0.077,0.029)	0.378	0.024 (−0.034,0.083)	0.417
	Tertile 3	0.031 (−0.030,0.093)	0.314	0.022 (−0.032,0.075)	0.432	0.076 (0.024,0.128)	0.004	0.002 (−0.059,0.064)	0.944	0.039 (−0.013,0.092)	0.143	0.071 (0.015,0.127)	0.014
	P for trend	0.388		0.433		0.004		0.955		0.137		0.013	
Model 2	Tertile 1	Reference	—	Reference	—	Reference	—	Reference	—	Reference	—	Reference	—
	Tertile 2	−0.26 (−0.082,0.030)	0.356	0.023 (−0.031,0.077)	0.403	0.038 (−0.016,0.092)	0.168	−0.037 (−0.094,0.019)	0.192	−0.018 (−0.072,0.035)	0.498	0.023 (−0.035,0.082)	0.434
	Tertile 3	0.033 (−0.028,0.095)	0.289	0.037 (−0.017,0.091)	0.176	0.077 (0.025,0.129)	0.004	0.003 (−0.059,0.064)	0.932	0.049 (−0.004,0.102)	0.067	0.068 (0.012,0.125)	0.018
	P for trend	0.352		0.177		0.004		0.973		0.064		0.016	
Model 3	Tertile 1	Reference	—	Reference	—	Reference	—	Reference	—	Reference	—	Reference	—
	Tertile 2	−0.024 (−0.080,0.032)	0.409	0.024 (−0.030,0.078)	0.389	0.042 (−0.012,0.097)	0.127	−0.032 (−0.088,0.024)	0.265	−0.015 (−0.067,0.038)	0.583	0.028 (−0.031,0.087)	0.356
	Tertile 3	0.034 (−0.028,0.095)	0.282	0.040 (−0.014,0.094)	0.143	0.076 (0.024,0.128)	0.004	0.009 (−0.053,0.071)	0.776	0.053 (0.001,0.105)	0.047	0.068 (0.011,0.124)	0.019
	P for trend	0.340		0.144		0.005		0.861		0.044		0.017	

Adjusted covariates:

Model 1 = age, sex, race.

Model 2 = Model 1 + (BMI, HDL-C, uric acid, waist circumference).

Model 3 = Model 2 + (abnormal otoscopy, abnormal tympanometry, present used of ototoxic agents, history of stroke, diabetes, heart disease).

Definition of abbreviations: High-PTA = Pure tone average at high frequencies, Low-PTA = Pure tone average at low frequencies, BMI = body mass index, HDL-C = high-density lipoprotein cholesterol.

Definition: Normal weight (BMI 18.5 to <25); overweight (BMI 25 to <30); obesity (BMI ≧ 30).
